# Predicting Hotspots and Prioritizing Protected Areas for Endangered Primate Species in Indonesia under Changing Climate

**DOI:** 10.3390/biology10020154

**Published:** 2021-02-15

**Authors:** Aryo Adhi Condro, Lilik Budi Prasetyo, Siti Badriyah Rushayati, I Putu Santikayasa, Entang Iskandar

**Affiliations:** 1 Tropical Biodiversity Conservation Program, Department of Forest Resources Conservation and Ecotourism, Faculty of Forestry, Kampus IPB Darmaga, IPB University (Bogor Agricultural University), Bogor 16680, Indonesia; aryo_acondro@apps.ipb.ac.id; 2 Department of Forest Resources Conservation and Ecotourism, Faculty of Forestry, Kampus IPB Darmaga, IPB University (Bogor Agricultural University), Bogor 16680, Indonesia; rushayati@apps.ipb.ac.id; 3 Department of Geophysics and Meteorology, Faculty of Mathematics and Natural Sciences, Kampus IPB Darmaga, IPB University (Bogor Agricultural University), Bogor 16680, Indonesia; ipsantika@apps.ipb.ac.id; 4 Primate Research Center, IPB University (Bogor Agricultural University), Jalan Lodaya II No 5, Bogor 16680, Indonesia; eiskandar@apps.ipb.ac.id

**Keywords:** species distribution model, primate conservation, Indonesia, Maxent, alpha diversity, climate change, protected areas, species richness

## Abstract

**Simple Summary:**

Primates play an essential role in human life and its ecosystem. However, Indonesian primates have suffered many threats due to climate change and altered landscapes that lead to extinction. Therefore, primate conservation planning and strategies are important in maintaining their population. We quantified how extensively the protected areas overlapped primate hotspots and how it changes under mitigation and worst-case scenarios of climate change. Finally, we provide protected areas recommendations based on species richness and land-use changes under the worst-case scenario for Indonesian primate conservation planning and management options.

**Abstract:**

Indonesia has a large number of primate diversity where a majority of the species are threatened. In addition, climate change is conservation issues that biodiversity may likely face in the future, particularly among primates. Thus, species-distribution modeling was useful for conservation planning. Herein, we present protected areas (PA) recommendations with high nature-conservation importance based on species-richness changes. We performed maximum entropy (Maxent) to retrieve species distribution of 51 primate species across Indonesia. We calculated species-richness change and range shifts to determine the priority of PA for primates under mitigation and worst-case scenarios by 2050. The results suggest that the models have an excellent performance based on seven different metrics. Current primate distributions occupied 65% of terrestrial landscape. However, our results indicate that 30 species of primates in Indonesia are likely to be extinct by 2050. Future primate species richness would be also expected to decline with the alpha diversity ranging from one to four species per 1 km^2^. Based on our results, we recommend 54 and 27 PA in Indonesia to be considered as the habitat-restoration priority and refugia, respectively. We conclude that species-distribution modeling approach along with the categorical species richness is effectively applicable for assessing primate biodiversity patterns.

## 1. Introduction

A recent assessment of global primate species showed that due to unsustainable human activities, more than half percent of primates are approaching extinction [1]. As one of the tropical mega-biodiversity countries, Indonesia has a large number of primate diversity [2] with the majority of the species being threatened (~83%; [3]). In 2017, Indonesia has 45 threatened primate species [4] and recently became larger based on new discoveries and biodiversity assessments [5,6,7,8]. Primates, which are one of the most crucial biodiversities in the tropical regions, are going to become extinct in the future due to habitat loss and climate change [4,9,10]. These species provide critical knowledge related to human evolution, biology, behaviors, and human health [1,11,12]. Furthermore, they are an essential component of the forest ecosystem because they help in the dispersal of seeds [11,13].

Climate-driven habitat degradation is one of the major conservation issues biodiversity will face in the future [14]. In combination, climate change and altered habitat would have detrimental effects for many wildlife species, potentially contributing to the extinction in the future [15,16,17,18] and also likely increase primate exposure to potentially harmful human-borne parasites, and vice versa [12]. Increase in temperature leads to faster reproduction and spread of parasites [4,19]. In addition, a primate-population pessimistic scenario of climate change shows that 72% of their primate population would be extinct in the future [4]. Despite the importance and vulnerability of this species, their distribution remains poorly known. Therefore, their potential response to environmental changes has not been evaluated. Such insight implies misleading primate conservation at the national scale [20]. Many other studies showed that the worst-case scenario of climate change is well represented in order to support conservation planning [21,22], and the majority of countries where primates occur (e.g., Brazil, Indonesia, Democratic Republic of Congo, and Madagascar) are suffering weak governance and consequently inefficient mitigation policies regarding climate change [2].

Climate change impacts on species distribution consist of: (i) dynamics in total suitable area, (ii) optimal environmental changes, and/or (iii) exposure to extinction [23]. To understand the consequences of climate change towards biodiversity, it is paramount that we can confidently assess the current and future potential distributions of species [24]. Also known as ecological-niche modeling (ENM), species-distribution modeling (SDM) uses relationships between occurrences of species and environmental conditions to predict species potential geographic distributions [25,26,27]. ENM basically has a stronger focus on predicting environmental parameters of fundamental ecological niches, whereas SDM is more focused on geographic distributions of species [28]. ENM and SDM have been broadly used for understanding species’ distributions under different spatiotemporal scenarios of environmental change via model transfer—e.g., climate change and altered habitat [29]. Species-distribution models have been widely used for various applications [30] at local, national, or regional scale [31,32,33]. This approach has been used to estimate the potential invasive species [34,35,36], to provide knowledge of the biology and biogeography of species [37], to identify biodiversity hotspots of threatened species [38,39], to assess conservation areas prioritization [31,40,41], and to discover new suitable habitat for species translocations [42]. Furthermore, SDM has also been broadly used to develop species richness using stacked-SDM methods [43] for identifying spatial diversity patterns regarding conservation-planning strategy [44]. Other essential components of biodiversity should also be included for protected-area management—e.g., endemism and species-conservation status [45]. In addition, a previous study stated that species richness and threatened species should be considered for managing protected areas and evaluating conservation effectiveness of protected areas [46,47]. Therefore, this study considered primate-richness-distribution changes within the protected areas using species-distribution modeling along with land-use dynamics to identify the prioritization of Indonesian primate conservation.

Herein, we present protected-areas recommendations with high nature-conservation importance (i.e., conservation priority and climate-refugia areas) based on novel updated species-richness pattern-changes information in order to contribute to the Indonesian primate conservation by modeling species distribution and richness changes towards primates in Indonesia in a complementary approach at the national level. To achieve our objectives, we independently modeled the spatial distribution of 51 threatened primate species at a high spatial resolution (30-arc second) using bioclimatic variables. We quantified how extensively the protected areas overlapped primate hotspots and how it changes under the worst-case scenario of climate change. Furthermore, we assessed potential candidates of protected areas priority and climate refugia by stacking species-distribution models.

## 2. Methods

### 2.1. Study Area

**Figure 1:**
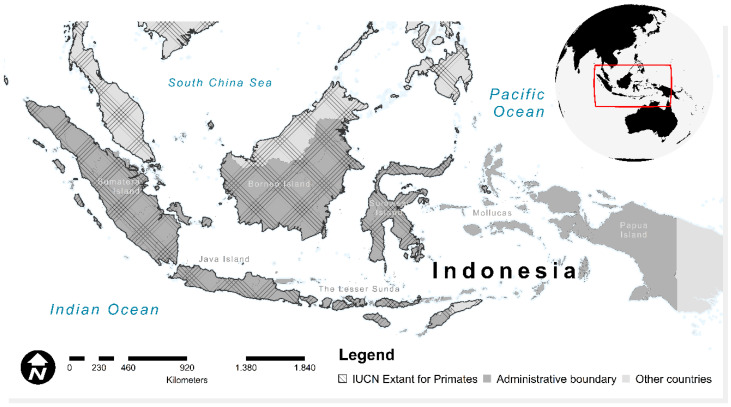
Extant ranges of primates in Indonesia. Hatched outline indicates the current known of potential biogeography of primates across the region (covered from the western to the central parts of Indonesia) based on The International Union for Conservation of Nature (IUCN) red list of threatened-species spatial database.

Indonesia is the largest maritime country with more than 17,000 islands [48], located in Southeast Asia (Figure 1). Straddling the equator, it has a warm, humid climate with an average temperature ranging from 21 to 31 °C, and rainfall, from 1780 to 3175 mm [49]. Therefore, almost all its regions are classified as tropical climate [50]. Due to its tropical conditions, it is one of the largest biodiversity nations in the world with a very high level of endemism, particularly in primate diversity, except in Mollucas Archipelago and Papua Island. Therefore, assessing the future redistribution of primate habitats is essential to carry out the conservation planning.

### 2.2. Occurrence Data

Primate occurrence data were obtained from the following sources: (1) Global Biodiversity Information Facility database (GBIF; [51]); (2) Citizen science database (iNaturalist; available from www.inaturalist.org/observations accessed on 10 November 2020); (3) IUCN extant species [3]; (4) fieldwork and monitoring project reports in Indonesia; and (5) scientific and reliable publications [52,53,54]. We used the keywords “Primates” and five family groups of primates found in Indonesia—i.e., “Cercopithecidae”, “Hominidae”, “Hylobatidae”, “Lorisidae”, and “Tarsidae”. We summarized the data from 1984–2020 with the basis of records from human observation and IUCN species-range maps for the species that were data deficient. The point of records was geopositioned (latitude–longitude coordinates) in decimal degrees based on the WGS 1984 datum. The occurrence, which had no additional information, including a relevant or detailed description, and duplicated data were excluded from the analysis.

Species with the total of records less than 10 occurrences were excluded from the analysis [10,11]. We performed a thinning technique using the Moran variogram to reduce autocorrelation and sampling bias effects in occurrence data provided by the spThin R package [55]. This study used a random partition of occurrences using K-folds by five-times folding for the model evaluation [56]. Afterwards, we collected 51 species of primates from 10 genera with the total occurrences of about 2469 point of records (Table S2). This study covered about 86% of the primate diversity in the Asia [57].

### 2.3. Environmental Covariates

In this study, we applied an Eltonian noise hypothesis perspective that neglecting the biotic factors (e.g., competitors, predators, and diseases) at large extents of the study area—i.e., maritime continents of Indonesia [58,59]. Climatic variables can be identified as a dominant control over the species distributions in regional to global scales [60]. Many previous studies also used climate-only variables for the species-distribution modeling—e.g., bioclimatic data [10,18,61]. For instance, we used 15 of the 19 variables of bioclimatic data to capture environmental covariates that represent a fundamental niche in the current condition, retrieved from WorldClim v.2.0 [62,63]. We excluded four covariates that combined precipitation and temperature information because they produced spatial artefacts [64,65]. To capture future climatic conditions, we used outputs from 19 global climate models (GCMs) based on CMIP5 data (Table S3)—i.e., coupled model intercomparison project phase 5, statistically downscaled using Delta methods [66]. We used mitigation and pessimistic greenhouse-gas-emission scenarios from representative concentration pathway (RCP4.5 and RCP8.5, [67]) for 2050 to explore future prioritization for primate conservation, also available at www.worldclim.org/data/v1.4/cmip5_30s (accessed on 10 November 2020). We performed an ensemble model to GCMs using simple average methods to obtain the most representative of the climate model [68].

The environmental covariates were used at a spatial resolution of 30 arc-second (~1 km) under both current and future conditions. Collinearity between the predictors can affect the estimation of model coefficients and interpretation of the model [69]. Therefore, we eliminated one of each pair of variables with |r| > 0.7 [70]. In this study, we did not find any issues in the degree of collinearity and collinearity shifting as well as after variables selection (Figure S1); thus, the data has a relatively good performance in model transferability [69]. Six uncorrelated predictors were used to calibrate models which consisted of mean diurnal range of temperature, temperature seasonality, minimum temperature of coldest month, temperature annual range, precipitation of wettest quarter, and precipitation of driest quarter (Table 1). 

### 2.4. Model Calibration and Evaluation

Maxent is one of the most broadly used algorithm in modeling species distribution [30,71]. Species-distribution models (SDMs) were performed using the maximum entropy (Maxent) in the maxnet R package [72]. We implemented default-tuned regularization values and all possible combinations featuring classes—i.e., linear (l), quadratic (q), product (p), threshold (t), and hinge (h) [73]. We used species-specific accessible area delimited as the movement component of the BAM diagram (biotic, abiotic, and movement) [58] based on the dispersal ability of the species information [74]. To assess the model transfer onto new conditions—i.e., projecting models on nonanalogous climates [75], we performed a mobility-oriented parity analysis [76]. Commonly, a model’s output represents the ecological niches (ENMs). To bring the ENMs closer to SDMs, we performed a posteriori methods based on occurrences–based restriction (OBR)—i.e., using the distance between points to exclude far suitable patches using MSDM R package [77]. 

To evaluate the models, we used five different metrics of model performance: area under the curve (AUC; [56]), Kappa coefficient [78], true skill statistic (TSS; [79]), Jaccard [80], and Sørensen [80]. Leroy et al. [80] showed that the use of TSS can be misleading towards model performance without a good quality of the occurrence-background data; thus, we also considered Jaccard and Sørensen indices to evaluate the model. For the evaluation, we converted continuous predicted probabilities into a binomial output using the suitability value that maximizes the TSS metric [79].

### 2.5. Biodiversity Redistribution and Protected-Area Prioritization

We calculated the potential range shifts of species-specific primate distributions based on the stacked binary maps of presence and absence produced from thresholding of continuous predictions [10,81]. The binary maps were also used to retrieve primate species richness across the study area with performing the accumulation of binomial outputs for all considered species in each cell (Equation (1)). The species richness of primates is calculated as the following equation:

(1) $$S\;=\;\overset{N}{\underset{i\;=\;1}{\sum }}{bin}_{i,k},$$

where *S* is the total number of species that occupied the cell, *N* is the total number of the species used in the analysis (*N* = 51), *bin* represents binomial output for each species, *i* and *k* are the indices for the species-specific and for the location of cell, respectively. Species richness is also known as the alpha diversity of primates [82].

The species-specific range shift of primates was calculated by the percentage of binomial outputs for a given primate species, comparing the current and future potential distribution (Equation (2)). Range shift is calculated as:

(2) $$R_{i}\;=\;{\left[\frac{\left(A_{t1}\;-\;A_{t0}\right)}{A_{t0}}\right]}_{i}\;\times \;100,$$

where *R_i_* represents the percent variation in the number of suitable cells for species-*i*; *A_t_*_1_ and *A_t_*_0_ are the total area of future potential distribution and current potential distribution for a given species. Negative value (*R_i_* < 0) and positive value (*R_i_* > 0) of range shifts represent the species contraction and species expansion, respectively.

We compiled the protected areas data within the study area from The World Database of Protected Areas (WPDA) by the United Nation Environment Programme World Conservation Monitoring Centre (UNEP WCMC; [83]). We used the terrestrial protected areas as the primate’s habitat, either national parks, nature reserves, wildlife sanctuaries, hunting parks, nature recreational parks, or pristine reserves [84]. Protected areas identified as having high conservation priority for primate corresponded to areas with susceptible to the species contraction due to climate change [85]. A prior study also considered the protected areas with the species that experienced a large proportional loss of suitable habitat as the highest-priority area [31]. 

**Figure 2:**
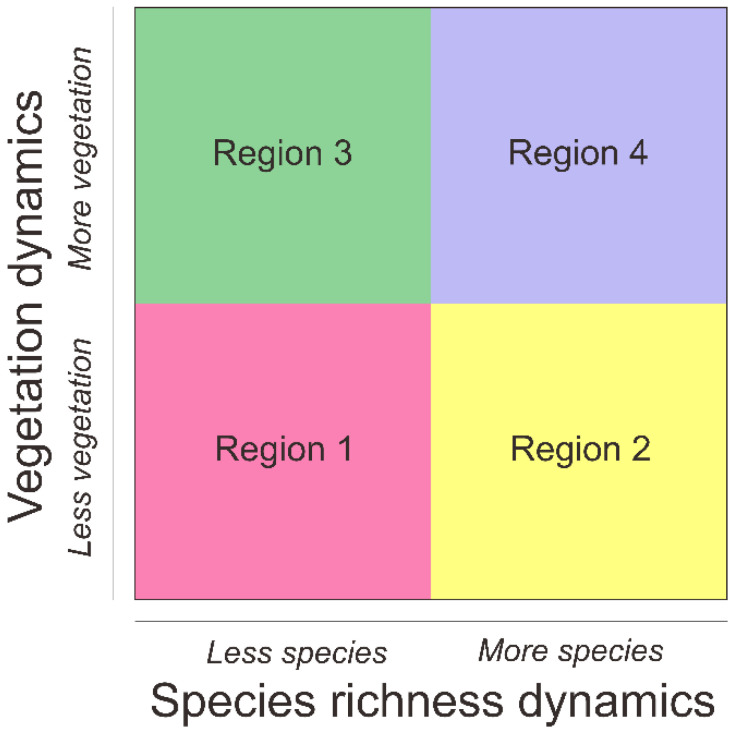
Conceptual diagram showing the intersection between the species richness and land-use dynamics for Indonesian primate conservation. Region 1: areas with less native vegetation and less species diversity in the future—habitat restoration; Region 2: areas with less native vegetation and more species diversity in the future—low priority by emphasizing habitat management; Region 3: areas with more native vegetation and less species diversity in the future—low priority by emphasizing species management; Region 4: areas with more native vegetation and more species diversity in the future—habitat refugia.

In this study, we considered the diversity changes of the primate species within the protected areas elaborated with the national land-use change dynamics as the component to determine the priority of the protected areas. We calculated the species-richness change (Δ*S* = *S_Future_*—*S_current_*; Equation (1)) to capture redistribution of Indonesian primate diversity due to climatic changing. We classified the species-richness change into two categories: values greater than zero and below zero that represent low and high risk of the species-richness change due to the climate shifting. Moreover, we classified the land-use map into two categories: “with less native-vegetation change” and “with more native-vegetation change” according to [86]. We performed land-use change simulation using the conversion of land use and its effects (CLUE-s) model [87,88] based on land-use maps from the Ministry of Environment and Forestry Indonesia [89] to retrieve land-use business-as-usual projection in 2050. We combined biophysical [90] and socioeconomic [91] parameters as the spatial determinants for changing land use in the study area [92]. Furthermore, we modified the conceptual framework from [86,93] to identify conservation planning and management options for Indonesian primates (Figure 2).

## 3. Results

### 3.1. Model Evaluation

The models were evaluated using five different metrics of model performance (Table S1). The results show the overall means of 0.93 for AUC (ranging from 0.61 to 0.99), 0.83 for Kappa (ranging from 0.56 to 0.99), 0.83 for TSS (ranging from 0.56 to 0.99), 0.85 for Jaccard (ranging from 0.68 to 0.99), and 0.91 for Sørensen (ranging from 0.79 to 0.98). Habitat suitability for the Mentawai langur (*Presbytis potenziani*) had the highest mean accuracy (AUC: 0.99; Kappa: 0.99; TSS: 0.99; Jaccard: 0.99; and Sørensen: 0.99) with standard error ranging from 0.000 to 0.003 for the used metrics. On the other hand, the Bornean white-bearded gibbon (*Hylobates albibarbis*) had the lowest mean accuracy (AUC: 0.61; Kappa: 0.56; TSS: 0.56; Jaccard: 0.68; and Sørensen: 0.79). However, most of the models for all species performed a relatively good performance with the metrics value were greater than 0.5.

The highest variable importance for most of the species was BIO6 (minimum temperature of the coldest month) and BIO16 (precipitation of the wettest quarter) with the variable-importance overall score average of about 49% for both variables. Further detail of the variable importance for each species can be seen in Figure S2.

### 3.2. Range Shifts of Primate Distribution under Climate Change

Generally, current potential distribution of primates (51 species) was occupied 65% of terrestrial landscape in Indonesia with the total area of about 1,426,472 km^2^. The species-specific current potential distributions of primates were ranging from the lowest 101 km^2^ to the highest 498,622 km^2^, which belong to *Tarsius tumpara* and *Nycticebus menagensis*, respectively. Primates potential distribution coverage in Indonesia consists of Sumatera Island, Borneo Island, Java Island, The Lesser Sunda, and Sulawesi Island. Moreover, we found a general range contraction of the future primates’ occupancy by 2050 with the total reduction of about 270,889 km^2^ and 282,117 km^2^ based on mitigation (RCP4.5) and business-as-usual (RCP8.5) scenarios, respectively. Future primates would be likely found in Sumatera Island, Borneo Island, the southern part of Sulawesi Island, and the northern part of Java Island.

**Figure 3:**
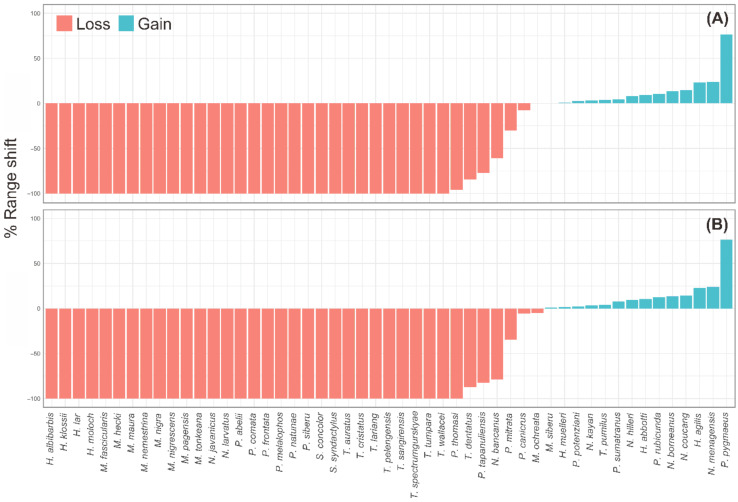
Range shift of potential distribution per species in response to the climate change for RCP4.5 scenario (**A**) and RCP8.5 scenario (**B**) for 2050. Range expansion is presented with the brick (red) color bar and the positive values. Range shrink or range contraction is presented with the turquoise color bar and the negative values.

Primates response to climate change varied among different species. We found that Indonesian primates will face a plenitude of effects of climate change on their geographic ranges. Our predictions indicate range contraction of potential distribution for most primate species, where 36 species will be expected to lose their habitats (*Range contraction_mean_* = −92% ± 4%) in the mitigation scenario and 37 species will be expected to lose their habitats (*Range contraction_mean_* = −93% ± 3%) in the business-as-usual scenario by 2050. Regarding its shifting, the results indicate that in both mitigation and BAU scenarios, 30 species of primates in Indonesia could possibly be extinct in the future by 2050 due to climate change. On the other hand, 15 species were predicted to have their potential distribution expanded (*Range expansion_mean_ =* 15% ± 5%), and 14 species were predicted to have their potential distribution expanded (*Range expansion_mean_ =* 13% ± 5%), based on RCP4.5 and RCP8.5 by 2050, respectively. This climatic shifting could thus lead the species to have more “winners” (i.e., whose potential distribution could expand) than “losers” [18]—see Figure 3. 

We found that 72% and 68% of the species from the old-world monkey family group (Cercopithecidae) could possibly be extinct based on mitigation and business-as-usual scenarios, respectively. Moreover, 75% species from Tarsidae family group will disappear by 2050 in both scenarios. The gibbon family group (Hylobatidae) will be extirpated by about 50% and 63% from their current distributions in RCP4.5 and RCP8.5 scenarios, respectively. Sumatran orangutan (*Pongo abelii*) and Javan slow loris (*Nycticebus javanicus*) would be expected to be extinct by 2050 in both scenarios. 

**Figure 4:**
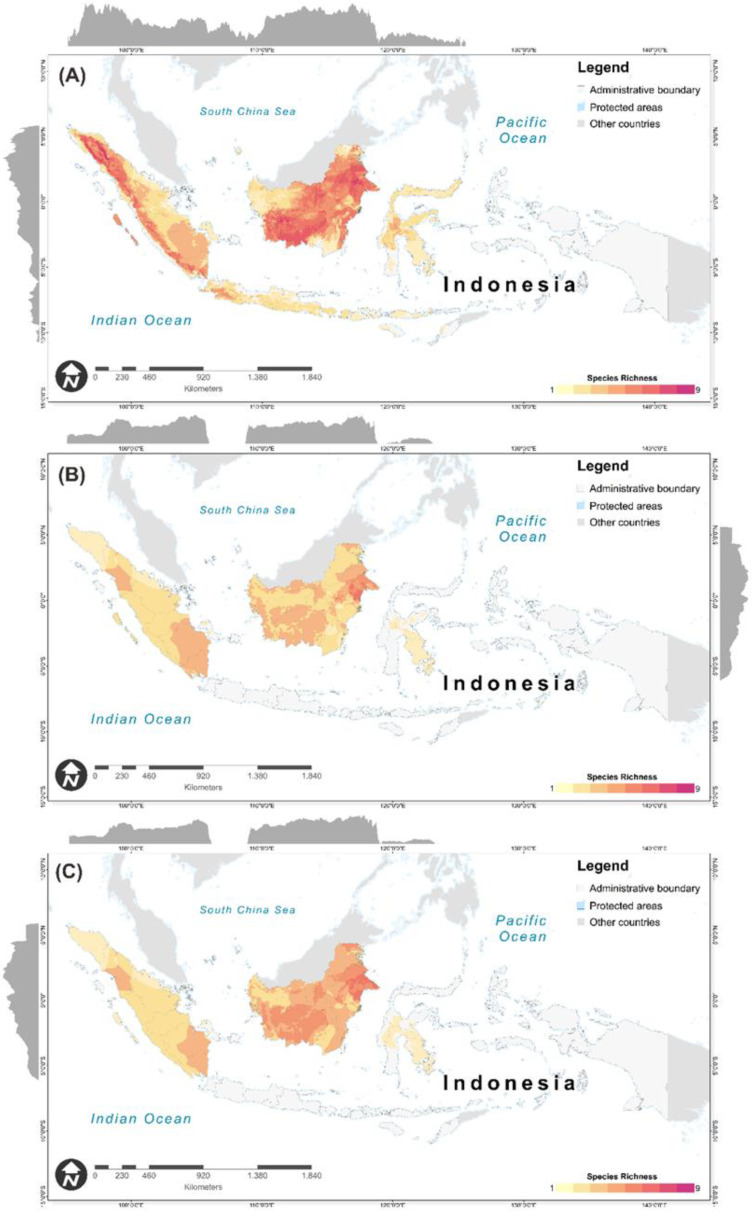
Species richness of Indonesian primates for current climate (**A**) and future climatic condition of RCP4.5 (**B**) and RCP8.5 (**C**) scenarios. the light blue outlined polygon indicates the protected areas of Indonesia. Darker red color indicates higher species richness, while pale yellow color indicates low species richness. Grey filled curves represent the richness distribution along latitudinal and longitudinal sections.

The results show that patterns of current primate species-richness varied among the regions, ranging from one to nine species per 1 km^2^. We found the primate-biodiversity hotspot along the Tropical Rainforest Heritage of Sumatera, which is concentrated in the northern part of Sumatera Island. In this area, eight to nine different species (e.g., *Symphalangus syndactylus*, *Trachypithecus cristatus*, *Hylobates lar*, *Macaca fascicularis*, *Macaca nemestrina*, *Nycticebus hilleri*, *Pongo abelii*, *Presbytis melalophos* (*sumatranus*), and *Presbytis thomasi*) can co-occur in the same cells that indicates highly diversity of primates in the area. Moreover, we also found high richness of primate species (~7 species) in the Kalimantan Island, particularly in the Heart of Borneo, which is the habitat for arboreal primate species. We also found the latitudinal and longitudinal variation in current primate species richness across Indonesia. The highest richness of primate-biodiversity hotspot was concentrated between 4° N to 5° N, which corresponds to the northern part of Sumatera Island landscapes. In the longitudinal section, we found the highest richness of primate species between 110° E to 120° E, which corresponds to the Kalimantan Island (Figure 4A).

Future primate species richness would be expected to decline by 2050, with the alpha diversity ranging from one to four species per 1 km^2^. We also found primate-diversity loss in the Lesser Sunda, Java Island, northern part of Sulawesi Island, and montane landscape of Sumatera Island. We can see richness reduction across the latitudinal and longitudinal sections, particularly in the northern part of Sumatera (95° E to 98° E and 4° N to 5° N) and also in the Kalimantan region (110° E to 120° E and 5° S to 5° S). We found the species richness decreasing from the current primate habitat with the total area of about 175,606 km^2^ and 198,741 km^2^ under mitigation and business-as-usual scenarios by 2050, respectively. 

### 3.3. Protected-Areas Prioritization for Primates under Worst-Case Scenario

Indonesia has a relatively enormous number of protected areas, covering from the western part to the eastern part of this country. Currently, Indonesia has 333 patches of protected areas, functioning in various ways for the extant primates with the total area of about 123,966.5 km^2^—i.e., 4% functioned as pristine reserves, 5% as wildlife sanctuaries, 1% as nature reserves, 1% as hunting parks, 47% as national parks, 2% as nature recreational parks, and 40% as unidentified protected areas. Current primate populations were spread over most of the Indonesian Islands, covering almost 50% of the protected areas within the extant primates. Nevertheless, species richness of primates would be declined by 2050 by about 41% in the future conditions. 

**Figure 5:**
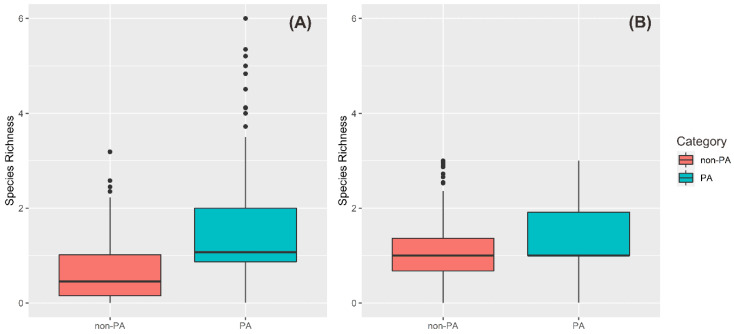
Species richness within protected areas/nonprotected areas in Indonesia of current (**A**) and future (**B**) conditions. PA: protected areas and non-PA: nonprotected areas.

Protected areas were significantly more effective in conserving the Indonesian primates than nonprotected areas based on their species richness (Kolmogorov–Smirnov two-sample test, current condition, *D* = 0.46, *p*-value < 0.001; future condition, *D* = 0.32, *p*-value < 0.01). We found primate species richness increases outside the protected areas in the future conditions by 2050. This result suggests range expansion of several primate species across the study area in the future (Figure 5).

**Figure 6:**
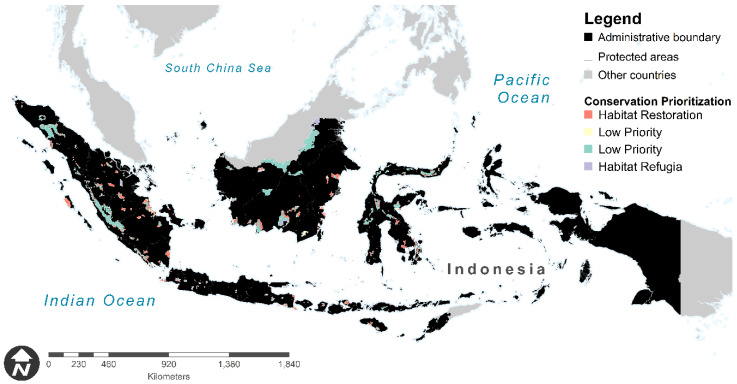
Conservation priority towards protected areas for primate diversity in Indonesia based on climate change and land use business-as-usual scenario. Red color shows Region 1 (i.e., areas with less native vegetation and less species diversity in the future—habitat restoration); pale yellow color represents Region 2 (i.e., areas with less native vegetation and more species diversity in the future—low priority by emphasizing habitat management); green shows Region 3 (i.e., areas with more native vegetation and less species diversity in the future—low priority by emphasizing species management); and purple color represents Region 4 (areas with more native vegetation and more species diversity in the future—habitat refugia).

We found a habitat-restoration prioritization (areas with less vegetation and less species in the future) within the protected areas in the future for Indonesian primate conservation, with a total area of about 47,235 km^2^ or covering 33% of the protected areas—e.g., Kerinci Seblat National Park, Siberut National Park, Tanjung Puting National Park, and Kutai National Park. Habitat refugia (areas with more vegetation and more species in the future) within the protected areas for future primate conservation was found mostly in Kalimantan, Sumatera, and Sulawesi regions, with a total area of about 8309 km^2^ or covered 6% of the entire protected areas—e.g., Kayan Mentarang National Park, Sebangau National Park, Kerumutan Wildlife Sanctuary, Danau Sentarum National Park, and Gunung Palung National Park. We also found about 5636 km^2^ (~4%) protected areas with less vegetation and more species in the future that suggested to low-priority areas by emphasizing habitat management. Moreover, the protected areas categorized as low-priority areas by emphasizing species management (more native vegetation and less species diversity in the future) have a total area of about 82,971 km^2^ or covered 58% of the protected areas (Figure 6). 

Recommendations for primate conservation within the protected areas can be seen in Table 2. Enhancing conservation management within the very high priority of protected areas—e.g., habitat restorations [86], patch-connectivity improvement [94], and protected-areas extension [46] should be carried out to maintain fitness of the primate species in Indonesia. However, we also recommend potential habitat refugia and restoration within the protected areas as potential habitat for primate species in the future.

## 4. Discussion

Conservation management towards protected areas to cope with the global climate change should be improved, particularly for endangered primate diversity in Indonesia. Species-distribution modeling (SDMs) or ecological-niche modeling (ENMs) should be considered to be used as essential tools for identifying the priority areas for effective biodiversity conservation [44,95]. Previous studies show a relatively good performance of climatic variables to capture biodiversity distributions over space and time—i.e., climate-only models [44,65]. Here, we apply the ENMs to the national-to-regional level by conducting species-specific distribution models for 51 endangered primate species using bioclimatic covariates to identify priority areas for primate conservation in Indonesia and produce the alpha diversity maps for focal Indonesian primates, using stacked-species-distribution models (S-SDM; [82]), also known as categorical species richness [81]. We considered only a worst-case scenario of climate change in order to bring out our main interest to provide up-stream planning towards primate conservation [21,22,96]. Many studies also used the S-SDM for prioritizing the conservation areas towards biodiversity [44,97]. Our results suggest that S-SDM using the Maxent algorithm has an excellent performance for each selected species of primate. Previous studies also suggest Maxent as a reliable tool to produce robust species-distribution models even with limited occurrence data [98]. There is a lack of research regarding S-SDM applications for Indonesian primates. Most of the Indonesian primate distribution studies were conducted by species-specific modeling [9,46,61,99,100]. Therefore, in this study, we provide reliable information regarding Indonesian primate diversity (multiple species) within the protected areas. This approach would be useful to identify sites for habitat conservation using species-specific-based habitat considerations, which are crucial when performing conservation management for multispecies.

The result suggests that most of the Indonesian primates had a significant dependence with the minimum temperature of the coldest month (mean overall importance of about 49%). Previous studies also show that minimum temperature strongly influenced the distribution of primates in the Neotropics, Africa, and Asia continents [96,101]. This study suggest that future mean annual temperature will likely have increases of about 1.40 °C from the current conditions. Temperature changes will lead to a broader dispersal capability in most of Cercopithecidae family groups that implies to the habitat shifting of the species [102]. Our results indicate more extreme precipitation in the future, which leads to increasing disease transmission to primates and food availability [103]. We also found that precipitation of the wettest quarter had a significant effect on several primate species distributions—e.g., *Tarsius spectrumgurskyae*, *Nasalis larvatus*, *Macaca nigra*, *Pongo pygmaeus*, and *Hylobates lar* (Figure S2). Current studies indicate the extremes in temperature and precipitation would significantly affect the primate risk [38]. Even the smallest amount of climate change expected for the tropics could therefore surpass the thermal tolerance of the species [104]. It follows that the extra amount of energy expended on homeostasis maintenance decreases the energy available for other functions, such as reproduction, which contributes to reduced fitness, loss of genetic diversity, and local extirpation [21,105]. Moreover, climatic shifting will be affecting the frugivorous and insectivorous species of primates due to their food availability being related to distributions of the fruit or insect development [101,106].

The redistribution of biodiversity with environmental, social, and economic consequences across the biosphere, particularly in Indonesia would be triggered by climate change [10,103]. Range shrink due to changing climate is a generally assumed pattern for primates [1]. In line with the previous study, our results indicate that most species (~86% of the Indonesian primate species used in this study) would exhibit range contractions that lead to extinction [107]. These range contractions may lead to local extinction [108] due to thermal physiological stress under climatic pressures [109]. Other study also shows range contractions towards Bornean orangutan by 2030 due to climate and land-cover changes [61]. In contrast with the previous study [61], our study suggests that Bornean orangutan (*Pongo pygmaeus*) would be expected to expand their range in response to climate change by 2050. Moreover, other studies revealed that Bornean orangutan had more adaptive capacity [110] and dispersal capability [111] than the other orangutan species—e.g., *Pongo abelii*. Furthermore, in this study, we found the Sumatran orangutan (*Pongo abelii*) would be expected to lose their suitable habitat in the future which would lead to extirpation of this species along with two vulnerable species, five endangered species, and two other critically endangered species. This study strongly suggests a high susceptibility to climate change in Indonesian primates.

Protected areas, widely recognized as the main strategy for biodiversity conservation [112], particularly for primates, have a greatly expanded coverage of ~12% of the terrestrial area of Indonesia [83]. Protected areas of Indonesia covered of about 71,732 km^2^ (~49%) primate distributions. Our findings confirm that the Indonesian protected areas still harbor a rich diversity of primates, including many keystone species—e.g., orangutan [100], slow loris [46], and gibbon [54]. Nevertheless, most of the Indonesian primates within the protected areas would be expected to suffer nonanalog climatic conditions by 2050 under the worst-case scenario. Moreover, we also found a larger area of primate distribution outside the protected areas (420,341 km^2^) than inside the protected areas. This result suggests that conservation strategies should be aimed at the outside protected areas as well—i.e., off-reserve management [113]. 

Conservation planning is an essential systematic processes [114]. Spatial-conservation strategy is supreme to develop a dynamic conservation under climatic shifting [40]. Most of the conservation strategies for protecting primate species under climate change should be directed at the species within the habitat at the highest risk. Moreover, identifying and protecting areas of climate refugia is supposed to reserve a larger number of species under climate change and to improve the natural adaptation [115]. We recommend 54 and 27 protected areas in Indonesia to be considered as the habitat restoration priority and refugia based on integrated climate and land-use dynamics, respectively (Table 2). The majority of habitat restoration and refugia function as a national park, which can be induced by anthropogenic activities within the areas. Our approach showed that only 6% of the national protected areas in Indonesia would be able to act as refugia for Indonesian primates. Furthermore, most species occur in high-risk areas. Indonesian primates can be impacted in various ways: land-use change (e.g., deforestation and agricultural expansion) which leads to range contraction [4,9]; climate shifting, which can cause redistribution of the species; behavior and physiology; and reproductive rate as well [38,116,117]. Managing species in the priority areas (i.e., high-risk) is more costly than using the refugia as the target habitat for future conservation [40]. Enhancing the connectivity of the landscape by restoring the habitat around the patches of protected areas is also important to improve range expansion of the primate species [94,118,119]. Moreover, the conservationist will only make the best decision if they can explicitly assess and identify the highest-priority conservation areas to avoid species extinctions [40].

## 5. Conclusions

In summary, our results suggest that species-distribution models have a good plausibility in capturing species-diversity patterns and their changes under climate change, particularly for Indonesian primates. Our results indicated that the species-distribution modeling approach can be used to provide reliable information for decision makers to consider whether the areas should be restored or purposed as refugia. This study provides fundamental primate-habitat conservation planning in Indonesia and also highlights species-richness hotspots and threat patterns of primate species, which allows for retrieving the potential areas for conservation actions. However, addressing the gaps in protected areas by creating the connectivity within the habitat patches will be crucial for biodiversity conservation of the tropical ecosystem in Indonesia.

## Figures and Tables

**Table 1:** Bioclimatic variables used in ecological-niche modeling of current and future potential distributions of primates in Indonesia.

Acronym	Description	Unit
Bio1	Annual mean temperature	°C
Bio2 ^2^	Annual mean diurnal range	°C
Bio3	Isothermality	%
Bio4 ^2^	Temperature seasonality	-
Bio5	Max temperature of warmest month	°C
Bio6 ^2^	Min temperature of coldest month	°C
Bio7 ^2^	Annual temperature range	°C
Bio8 ^1^	Mean temperature of wettest quarter	°C
Bio9 ^1^	Mean temperature of driest quarter	°C
Bio10	Mean temperature of warmest quarter	°C
Bio11	Mean temperature of coldest quarter	°C
Bio12	Annual precipitation	mm
Bio13	Precipitation of wettest month	mm
Bio14	Precipitation of driest month	mm
Bio15	Precipitation seasonality	%
Bio16 ^2^	Precipitation of wettest quarter	mm
Bio17 ^2^	Precipitation of driest quarter	mm
Bio18 ^1^	Precipitation of warmest quarter	mm
Bio19 ^1^	Precipitation of coldest quarter	mm

^1^ Covariates excluded from the analysis due to its odd spatial anomalies. ^2^ Six variables were selected for modeling.

**Table 2:** Recommendations for habitat restoration and refugia within protected areas for Kalimantan, Java, Sulawesi, and Sumatera Islands based on the species richness and habitat changes.

Region	Recommendations for Habitat Restoration	Recommendations for Habitat Refugia
Kalimantan	Tanjung Puting Kutai Sebangau Muara Kaman Sedulang Bukit Soeharto Sungai Kapuas Teluk Adang Teluk Apar Lamandau Sungai Barito Tanjung Malatayur Teluk Pamukan	Kayan Mentarang Sebangau Danau Sentarum Gunung Palung Gunung Nyiut Penrissen Sultan Adam Gunung Melintang Kutai Muara Kaman Sedulang Gunung Asuansang Bukit Baka—Bukit Raya Gunung Dako Betung Kerihun Gunung Raya Passi
Java and the Lesser Sunda	Gunung Tambora Selatan Manupeu Tanadaru Laiwangi Wanggameti Gunung Rinjani Bali Barat Alas Purwo Gunung Halimun—Salak Ujung Kulon Meru Betiri Baluran Bromo Tengger Semeru Gunung Gede—Pangrango Gunung Simpang Gunung Masigit Kareumbi Gunung Ciremai	-
Sumatera	Kerinci Seblat Siberut Bukit Barisan Selatan Way Kambas Gunung Leuser Bukit Tiga Puluh Tesso Nilo Berbak Bukit Rimbang Bukit Baling Rawa Singkil Bukit Dua Belas Dangku Gunung Raya Padang Sugihan Arau Hilir and Air Terusan Zamrud	Kerumutan Batang Gadis Pulau Pini Tasik Serkap-Tasik Sarang Burung Sembilang Bukit Rimbang Bukit Baling Tasik Besar-Tasik Metas Tasik Belat
Sulawesi	Rawa Aopa Watumohai Lore Lindu Bogani Nani Wartabone Danau Towuti Bantimurung Tanjung Peropa Danau Matano Faruhumpenai Lambusango Panua Kepulauan Togean	Buton Utara Morowali Gunung Dako Ganda Dewata

## Data Availability

Bioclimatic data used in this study can be downloaded at https://www.worldclim.org/. Primates occurrences were collected on freely available web sources, listed in Methods section, Occurrence Data sub-section. High-resolution raster data should be obtained upon email request to the corresponding author.

## Supplementary Materials

The following are available online at https://www.mdpi.com/2079-7737/10/2/154/s1, Figure S1: Degree of collinearity for the model training under current (A) and future (B) climate conditions using variables selection (|r| ≤ 0.7). Collinearity shift between training and projected environments (C). In this study, we did not find any issues in the degree of collinearity for each climatic conditions and collinearity shift after variables selection, Figure S2: Variable-importance (VI) scores for the potential distribution model of each Primates species. Big and red to yellow circles indicate a higher dependence of the species occurrence on the corresponding bioclimatic covariate. In the other hand, small and purple to blue circles indicate low dependence. The environmental covariates used in the models were, BIO16: precipitation of the wettest quarter, BIO17: precipitation of the driest quarter, BIO2: annual mean diurnal range of temperature, BIO4: temperature seasonality, BIO6: minimum temperature of the coldest month, and BIO7: annual temperature range, Table S1: Predictive performance of the models on the k-fold (k = 5) for each species using five different metrics of accuracy, show in mean of value ± SE—i.e., Area Under the ROC Curve (AUC), Kappa coefficient, true skill statistic (TSS), Jaccard index, and Sørensen index, Table S2: Species-specific information of primate occurrences across Indonesia as response variable in the species distribution models. VU: Vulnerable; EN: Endangered; and CR: Critically endangered, Table S3: Global climate models used in ecological-niche-modeling projections for 2050 based on RCP4.5 and RCP8.5 scenarios.

## References

[1] Estrada A., Garber P.A., Rylands A.B., Roos C., Fernandez-Duque E., Di Fiore A., Anne-Isola Nekaris K., Nijman V., Heymann E.W., Lambert J.E. (2017). Impending extinction crisis of the world’s primates: Why primates matter. Sci. Adv..

[2] Grow N., Gursky-Doyen S., Supriatna J., Gursky-Doyen S., Supriatna J. (2010). Introduction. Indonesian Primates.

[3] (2012). IUCN Red List Categories and Criteria: Version 3.1.

[4] Estrada A., Garber P.A., Mittermeier R.A., Wich S., Gouveia S., Dobrovolski R., Nekaris K.A.I., Nijman V., Rylands A.B., Maisels F. (2018). Primates in peril: The significance of Brazil, Madagascar, Indonesia and the Democratic Republic of the Congo for global primate conservation. PeerJ.

[5] Nekaris K.A.I., Miard P. (2020). *Nycticebus kayan*, Kayan Slow Loris. IUCN Red List of Threatened Species.

[6] Quinten M., Setiawan A., Cheyne S., Traeholt C., Whittaker D. (2020). *Simias concolor*, Pig-tailed Snub-nosed Langur. IUCN Red List of Threatened Species.

[7] Cheyne S., Setiawan A., Traeholt C. (2020). *Presbytis canicrus*, Miller’s Grizzled Langur. IUCN Red List of Threatened Species.

[8] Nowak M.G., Rianti P., Wich S.A., Meijaard E., Fredriksson G. (2020). *Pongo tapanuliensis*, Tapanuli Orangutan. IUCN Red List of Threatened Species.

[9] Wich S.A., Singleton I., Nowak M.G., Atmoko S.S.U., Nisam G., Arif S.M., Putra R.H., Ardi R., Fredriksson G., Usher G. (2016). Land-cover changes predict steep declines for the Sumatran orangutan (*Pongo abelii*). Sci. Adv..

[10] Sales L.P., Ribeiro B.R., Pires M.M., Chapman C.A., Loyola R. (2019). Recalculating route: Dispersal constraints will drive the redistribution of Amazon primates in the Anthropocene. Ecography.

[11] Chapman C.A., Bonnell T.R., Gogarten J.F., Lambert J.E., Omeja P.A., Twinomugisha D., Wasserman M.D., Rothman J.M. (2013). Are Primates Ecosystem Engineers?. Int. J. Primatol..

[12] Olival K.J., Hosseini P.R., Zambrana-Torrelio C., Ross N., Bogich T.L., Daszak P. (2017). Host and viral traits predict zoonotic spillover from mammals. Nature.

[13] Razafindratsima O.H., Sato H., Tsuji Y., Culot L. (2018). Advances and Frontiers in Primate Seed Dispersal. Int. J. Primatol..

[14] Buse J., Boch S., Hilgers J., Griebeler E.M. (2015). Conservation of threatened habitat types under future climate change—Lessons from plant-distribution models and current extinction trends in southern Germany. J. Nat. Conserv..

[15] Chen I.C., Hill J.K., Ohlemüller R., Roy D.B., Thomas C.D. (2011). Rapid range shifts of species associated with high levels of climate warming. Science.

[16] Struebig M.J., Fischer M., Gaveau D.L.A., Meijaard E., Wich S.A., Gonner C., Sykes R., Wilting A., Kramer-Schadt S. (2015). Anticipated climate and land-cover changes reveal refuge areas for Borneo’s orang-utans. Glob. Chang. Biol..

[17] Gouveia S.F., Souza-Alves J.P., Rattis L., Dobrovolski R., Jerusalinsky L., Beltrão-Mendes R., Ferrari S.F. (2016). Climate and land use changes will degrade the configuration of the landscape for titi monkeys in eastern Brazil. Glob. Chang. Biol..

[18] Sales L., Ribeiro B.R., Chapman C.A., Loyola R. (2020). Multiple dimensions of climate change on the distribution of Amazon primates. Perspect. Ecol. Conserv..

[19] Barrett M.A., Brown J.L., Junge R.E., Yoder A.D. (2013). Climate change, predictive modeling and lemur health: Assessing impacts of changing climate on health and conservation in Madagascar. Biol. Conserv..

[20] De Grammont P.C., Cuarón A.D. (2006). An evaluation of threatened species categorization systems used on the american continent. Conserv. Biol..

[21] Ribeiro B.R., Sales L.P., De Marco P., Loyola R. (2016). Assessing mammal exposure to climate change in the Brazilian Amazon. PLoS ONE.

[22] Lehmann J., Korstjens A.H., Dunbar R.I.M. (2010). Apes in a changing world—the effects of global warming on the behaviour and distribution of African apes. J. Biogeogr..

[23] Garcia R.A., Cabeza M., Rahbek C., Araújo M.B. (2014). Multiple dimensions of climate change and their implications for biodiversity. Science.

[24] Beaumont L.J., Hughes L., Poulsen M. (2005). Predicting species distributions: Use of climatic parameters in BIOCLIM and its impact on predictions of species’ current and future distributions. Ecol. Model..

[25] Feng X., Park D.S., Walker C., Peterson A.T., Merow C., Papeş M. (2019). A checklist for maximizing reproducibility of ecological niche models. Nat. Ecol. Evol..

[26] Franklin J. (2010). Mapping Species Distributions: Spatial Inference and Prediction.

[27] Guisan A., Zimmermann N.E. (2000). Predictive habitat distribution models in ecology. Ecol. Model..

[28] Peterson A.T., Soberón J. (2012). Species distribution modeling and ecological niche modeling: Getting the Concepts Right. Nat. Conserv..

[29] Werkowska W., Márquez A.L., Real R., Acevedo P. (2017). A practical overview of transferability in species distribution modeling. Environ. Rev..

[30] Araújo M.B., Anderson R.P., Barbosa A.M., Beale C.M., Dormann C.F., Early R., Garcia R.A., Guisan A., Maiorano L., Naimi B. (2019). Standards for distribution models in biodiversity assessments. Sci. Adv..

[31] Kremen C., Cameron A., Moilanen A., Phillips S.J., Thomas C.D., Beentje H., Dransfield J., Fisher B.L., Glaw F., Good T.C. (2008). Aligning conservation priorities across taxa in Madagascar with high-resolution planning tools. Science.

[32] Faleiro F.V., Machado R.B., Loyola R.D. (2013). Defining spatial conservation priorities in the face of land-use and climate change. Biol. Conserv..

[33] Araújo M.B., Alagador D., Cabeza M., Nogués-Bravo D., Thuiller W. (2011). Climate change threatens European conservation areas. Ecol. Lett..

[34] Sales L.P., Ribeiro B.R., Hayward M.W., Paglia A., Passamani M., Loyola R. (2017). Niche conservatism and the invasive potential of the wild boar. J. Anim. Ecol..

[35] Tingley R., Vallinoto M., Sequeira F., Kearney M.R. (2014). Realized niche shift during a global biological invasion. Proc. Natl. Acad. Sci. USA.

[36] Broennimann O., Treier U.A., Müller-Schärer H., Thuiller W., Peterson A.T., Guisan A. (2007). Evidence of climatic niche shift during biological invasion. Ecol. Lett..

[37] Melo-Merino S.M., Reyes-Bonilla H., Lira-Noriega A. (2020). Ecological niche models and species distribution models in marine environments: A literature review and spatial analysis of evidence. Ecol. Model..

[38] Graham T.L., Matthews H.D., Turner S.E. (2016). A Global-Scale Evaluation of Primate Exposure and Vulnerability to Climate Change. Int. J. Primatol..

[39] Saarimaa M., Aapala K., Tuominen S., Karhu J., Parkkari M., Tolvanen A. (2019). Predicting Hotspots for Threatened Plant Species in Boreal Peatlands. Biodivers. Conserv..

[40] Ribeiro B.R., Sales L.P., Loyola R. (2018). Strategies for mammal conservation under climate change in the Amazon. Biodivers. Conserv..

[41] Fuller C., Ondei S., Brook B.W., Buettel J.C. (2020). Protected-area planning in the Brazilian Amazon should prioritize additionality and permanence, not leakage mitigation. Biol. Conserv..

[42] Jovanovic T., Arnold R., Booth T. (2000). Determining the climatic suitability of Eucalyptus dunnii for plantations in Australia, China and Central and South America. New For..

[43] Calabrese J.M., Certain G., Kraan C., Dormann C.F. (2014). Stacking species distribution models and adjusting bias by linking them to macroecological models. Glob. Ecol. Biogeogr..

[44] Koch R., Almeida-Cortez J.S., Kleinschmit B. (2017). Revealing areas of high nature conservation importance in a seasonally dry tropical forest in Brazil: Combination of modelled plant diversity hot spots and threat patterns. J. Nat. Conserv..

[45] Fleishman E., Noss R.F., Noon B.R. (2006). Utility and limitations of species richness metrics for conservation planning. Ecol. Indic..

[46] Thorn J.S., Nijman V., Smith D., Nekaris K.A.I. (2009). Ecological niche modelling as a technique for assessing threats and setting conservation priorities for Asian slow lorises (Primates: Nycticebus). Divers. Distrib..

[47] Chape S., Harrison J., Spalding M., Lysenko I. (2005). Measuring the extent and effectiveness of protected areas as an indicator for meeting global biodiversity targets. Philos. Trans. R. Soc. B Biol. Sci..

[48] Cleary D.F.R., De Vantier L. (2019). Indonesia: Threats to the Country’s Biodiversity.

[49] Mitchell T.D., Jones P.D. (2005). An improved method of constructing a database of monthly climate observations and associated high-resolution grids. Int. J. Climatol..

[50] Peel M.C., Finlayson B.L., McMahon T.A. (2007). Updated world map of the Köppen-Geiger climate classification. Hydrol. Earth Syst. Sci..

[51] GBIF Global Biodiversity Information Facility. https://www.gbif.org/.

[52] Singleton I., Wich S.A., Nowak M., Usher G., Utami-Atmoko S.S. (2017). *Pongo abelii*, Sumatran Orangutan. IUCN Red List of Threatened Species.

[53] Setiawan A., Traeholt C. (2020). *Presbytis thomasi*, Thomas Langur. IUCN Red List of Threatened Species.

[54] Nijman V. (2020). *Hylobates moloch*, Silvery Gibbon. IUCN Red List of Threatened Species.

[55] Aiello-Lammens M.E., Boria R.A., Radosavljevic A., Vilela B., Anderson R.P. (2015). spThin: An R package for spatial thinning of species occurrence records for use in ecological niche models. Ecography.

[56] Fielding A.H., Bell J.F. (1997). A review of methods for the assessment of prediction errors in conservation presence/absence models. Environ. Conserv..

[57] Lehman S.M., Fleagle J.G., Lehman S.M., Fleagle J.G. (2006). Primate Biogeography: A Review. Primate Biogeography.

[58] Soberon J., Peterson A.T. (2005). Interpretation of Models of Fundamental Ecological Niches and Species’ Distributional Areas. Biodivers. Inform..

[59] Soberón J., Nakamura M. (2009). Niches and distributional areas: Concepts, methods, and assumptions. Proc. Natl. Acad. Sci. USA.

[60] Willis K.J., Whittaker R.J. (2002). Species diversity—Scale matters. Science.

[61] Condro A.A., Prasetyo L.B., Rushayati S.B. Short-term projection of Bornean orangutan spatial distribution based on climate and land cover change scenario. Proceedings of the Sixth International Symposium on LAPAN-IPB Satellite.

[62] Fick S.E., Hijmans R.J. (2017). WorldClim 2: New 1-km spatial resolution climate surfaces for global land areas. Int. J. Climatol..

[63] Hijmans R.J., Cameron S.E., Parra J.L., Jones P.G., Jarvis A. (2005). Very high resolution interpolated climate surfaces for global land areas. Int. J. Climatol..

[64] Escobar L.E., Lira-Noriega A., Medina-Vogel G., Townsend Peterson A. (2014). Potential for spread of the white-nose fungus (*Pseudogymnoascus destructans*) in the Americas: Use of Maxent and NicheA to assure strict model transference. Geospat. Health.

[65] Marques R., Krüger R.F., Peterson A.T., De Melo L.F., Vicenzi N., Jiménez-García D. (2020). Climate change implications for the distribution of the babesiosis and anaplasmosis tick vector, Rhipicephalus (Boophilus) microplus. Vet. Res..

[66] Navarro-Racines C., Tarapues J., Thornton P., Jarvis A., Ramirez-Villegas J. (2020). High-resolution and bias-corrected CMIP5 projections for climate change impact assessments. Sci. Data.

[67] van Vuuren D.P., Edmonds J., Kainuma M., Riahi K., Thomson A., Hibbard K., Hurtt G.C., Kram T., Krey V., Lamarque J.F. (2011). The representative concentration pathways: An overview. Clim. Chang..

[68] Buisson L., Thuiller W., Casajus N., Lek S., Grenouillet G. (2010). Uncertainty in ensemble forecasting of species distribution. Glob. Chang. Biol..

[69] Feng X., Park D.S., Liang Y., Pandey R., Papeş M. (2019). Collinearity in ecological niche modeling: Confusions and challenges. Ecol. Evol..

[70] Dormann C.F., Elith J., Bacher S., Buchmann C., Carl G., Carré G., Marquéz J.R.G., Gruber B., Lafourcade B., Leitão P.J. (2013). Collinearity: A review of methods to deal with it and a simulation study evaluating their performance. Ecography.

[71] Elith J., Phillips S.J., Hastie T., Dudík M., Chee Y.E., Yates C.J. (2011). A statistical explanation of MaxEnt for ecologists. Divers. Distrib..

[72] Phillips S. Maxnet: Fitting ‘Maxent’ Species Distribution Models with “glmnet”. https://rdrr.io/cran/maxnet/.

[73] Phillips S.J., Anderson R.P., Dudík M., Schapire R.E., Blair M.E. (2017). Opening the black box: An open-source release of Maxent. Ecography.

[74] Barve N., Barve V., Jiménez-Valverde A., Lira-Noriega A., Maher S.P., Peterson A.T., Soberón J., Villalobos F. (2011). The crucial role of the accessible area in ecological niche modeling and species distribution modeling. Ecol. Model..

[75] Fitzpatrick M.C., Blois J.L., Williams J.W., Nieto-Lugilde D., Maguire K.C., Lorenz D.J. (2018). How will climate novelty influence ecological forecasts? Using the Quaternary to assess future reliability. Glob. Chang. Biol..

[76] Owens H.L., Campbell L.P., Dornak L.L., Saupe E.E., Barve N., Soberón J., Ingenloff K., Lira-Noriega A., Hensz C.M., Myers C.E. (2013). Constraints on interpretation of ecological niche models by limited environmental ranges on calibration areas. Ecol. Model..

[77] Mendes P., Velazco S.J.E., de Andrade A.F.A., De Marco P. (2020). Dealing with overprediction in species distribution models: How adding distance constraints can improve model accuracy. Ecol. Model..

[78] Cohen J. (1960). A Coefficient of Agreement for Nominal Scales. Educ. Psychol. Meas..

[79] Allouche O., Tsoar A., Kadmon R. (2006). Assessing the accuracy of species distribution models: Prevalence, kappa and the true skill statistic (TSS). J. Appl. Ecol..

[80] Leroy B., Delsol R., Hugueny B., Meynard C.N., Barhoumi C., Barbet-Massin M., Bellard C. (2018). Without quality presence–absence data, discrimination metrics such as TSS can be misleading measures of model performance. J. Biogeogr..

[81] Alaniz A.J., Carvajal M.A., Vergara P.M., Fierro A., Moreira-Arce D., Rojas-Osorio A., Soto G.E., Rodewald A.D. (2020). Trophic behavior of specialist predators from a macroecological approach: The case of the magellanic woodpecker in south American temperate forests. Glob. Ecol. Conserv..

[82] Mateo R.G., Felicísimo Á.M., Pottier J., Guisan A., Muñoz J. (2012). Do stacked species distribution models reflect altitudinal diversity patterns?. PLoS ONE.

[83] UNEP The World Database of Protected Areas. https://www.protectedplanet.net/country/IDN.

[84] Rahman D.A., Condro A.A., Rianti P., Masy’ud B., Aulagnier S., Semiadi G. (2020). Geographical analysis of the Javan deer distribution in Indonesia and priorities for landscape conservation. J. Nat. Conserv..

[85] Haight J., Hammill E. (2020). Protected areas as potential refugia for biodiversity under climatic change. Biol. Conserv..

[86] Borges F.J.A., Loyola R. (2020). Climate and land-use change refugia for Brazilian Cerrado birds. Perspect. Ecol. Conserv..

[87] Verburg P.H., Soepboer W., Veldkamp A., Limpiada R., Espaldon V., Mastura S.S.A. (2002). Modeling the spatial dynamics of regional land use: The CLUE-S model. Environ. Manag..

[88] Verburg P.H., Overmars K.P. (2009). Combining top-down and bottom-up dynamics in land use modeling: Exploring the future of abandoned farmlands in Europe with the Dyna-CLUE model. Landsc. Ecol..

[89] MoEF Land Cover Maps of Indonesia. http://webgis.menlhk.go.id:8080/pl/pl.htm.

[90] Farr T.G., Rosen P.A., Caro E., Crippen R., Duren R., Hensley S., Kobrick M., Paller M., Rodriguez E., Roth L. (2007). The shuttle radar topography mission. Rev. Geophys..

[91] Weiss D.J., Nelson A., Gibson H.S., Temperley W., Peedell S., Lieber A., Hancher M., Poyart E., Belchior S., Fullman N. (2018). A global map of travel time to cities to assess inequalities in accessibility in 2015. Nature.

[92] Verburg P.H., Ritsema van Eck J.R., de Nijs T.C.M., Dijst M.J., Schot P. (2004). Determinants of land-use change patterns in the Netherlands. Environ. Plan. B Plan. Des..

[93] Cabral Rezende G., Sobral-Souza T., Culot L. (2020). Integrating climate and landscape models to prioritize areas and conservation strategies for an endangered arboreal primate. Am. J. Primatol..

[94] Hodgson J.A., Wallis D.W., Krishna R., Cornell S.J. (2016). How to manipulate landscapes to improve the potential for range expansion. Methods Ecol. Evol..

[95] Graham V., Baumgartner J.B., Beaumont L.J., Esperón-Rodríguez M., Grech A. (2019). Prioritizing the protection of climate refugia: Designing a climate-ready protected area network. J. Environ. Plan. Manag..

[96] Carvalho J.S., Graham B., Rebelo H., Bocksberger G., Meyer C.F.J., Wich S., Kühl H.S. (2019). A global risk assessment of primates under climate and land use/cover scenarios. Glob. Chang. Biol..

[97] Zellmer A.J., Claisse J.T., Williams C.M., Schwab S., Pondella D.J. (2019). Predicting optimal sites for ecosystem restoration using stacked-species distribution modeling. Front. Mar. Sci..

[98] Pearson R.G., Raxworthy C.J., Nakamura M., Townsend Peterson A. (2007). Predicting species distributions from small numbers of occurrence records: A test case using cryptic geckos in Madagascar. J. Biogeogr..

[99] Iwanda R., Prasetyo L.B., Rinaldi D., Pairah P., Septiana W., Erlan M., Hilmy Y. Priority restoration area mapping of Javan Gibbon Habitat (Hylobates moloch Audebert 1798) in Gunung Halimun Salak National Park as a result of global climate change. Proceedings of the Sixth International Symposium on LAPAN-IPB Satellite.

[100] Wich S.A., Meijaard E., Marshall A.J., Husson S., Ancrenaz M., Lacy R.C., Van Schaik C.P., Sugardjito J., Simorangkir T., Traylor-Holzer K. (2008). Distribution and conservation status of the orang-utan (*Pongo* spp.) on Borneo and Sumatra: How many remain?. Oryx.

[101] Monroe B.P., Nakazawa Y.J., Reynolds M.G., Carroll D.S. (2014). Estimating the geographic distribution of human Tanapox and potential reservoirs using ecological niche modeling. Int. J. Health Geogr..

[102] Brandon-Jones D. (1996). The Asian Colobinae (Mammalia: Cercopithecidae) as indicators of quaternary climatic change. Biol. J. Linn. Soc..

[103] Pecl G.T., Araújo M.B., Bell J.D., Blanchard J., Bonebrake T.C., Chen I.C., Clark T.D., Colwell R.K., Danielsen F., Evengård B. (2017). Biodiversity redistribution under climate change: Impacts on ecosystems and human well-being. Science.

[104] Bennett J.M., Calosi P., Clusella-Trullas S., Martínez B., Sunday J., Algar A.C., Araújo M.B., Hawkins B.A., Keith S., Kühn I. (2018). GlobTherm, a global database on thermal tolerances for aquatic and terrestrial organisms. Sci. Data.

[105] Khaliq I., Hof C., Prinzinger R., Böhning-Gaese K., Pfenninger M. (2014). Global variation in thermal tolerances and vulnerability of endotherms to climate change. Proc. R. Soc. B Biol. Sci..

[106] Korstjens A.H., Hillyer A., Wich S.A., Marshall A.J. (2016). Primates and climate change: A review of current knowledge. An Introduction to Primate Conservation.

[107] Dirzo R., Young H.S., Galetti M., Ceballos G., Isaac N.J.B., Collen B. (2014). Defaunation in the Anthropocene. Science.

[108] Urban M.C. (2015). Accelerating extinction risk from climate change. Sci. Rep..

[109] Dillon M.E., Wang G., Huey R.B. (2010). Global metabolic impacts of recent climate warming. Nature.

[110] Marshall A.J., Lacy R., Ancrenaz M., Byers O., Husson S.J., Leighton M., Meijaard E., Rosen N., Singleton I., Stephens S. (2009). Orangutan population biology, life history, and conservation: Perspectives from population viability analysis models. Orangutans Geogr. Var. Behav. Ecol. Conserv..

[111] Singleton I., Knott C.D., Morrogh-Bernard H.C., Wich S.A., Van Schaik C.P. (2009). Ranging behavior of orangutan females and social organization. Orangutans Geogr. Var. Behav. Ecol. Conserv..

[112] Xavier da Silva M., Paviolo A., Tambosi L.R., Pardini R. (2018). Effectiveness of Protected Areas for biodiversity conservation: Mammal occupancy patterns in the Iguaçu National Park, Brazil. J. Nat. Conserv..

[113] International Bioversity Conservation Outside Protected Areas. http://www.cropwildrelatives.org/resources/in-situ-conservation-manual/.

[114] Margules C.R., Pressey R.L. (2000). Systematic conservation planning. Nature.

[115] Game E.T., Lipsett-Moore G., Saxon E., Peterson N., Sheppard S. (2011). Incorporating climate change adaptation into national conservation assessments. Glob. Chang. Biol..

[116] Gaveau D.L.A.A., Wich S., Epting J., Juhn D., Kanninen M., Leader-williams N. (2009). The future of forests and orangutans (Pongoabelii) in Sumatra: Predicting impacts of oil palm plantations, road construction, and mechanisms for reducing carbon emissions from deforestation. Environ. Res. Lett..

[117] Wich S.A., Fredriksson G.M., Usher G., Peters H.H., Priatna D., Basalamah F., Susanto W., Kühl H. (2012). Hunting of Sumatran orang-utans and its importance in determining distribution and density. Biol. Conserv..

[118] Hodgson J.A., Thomas C.D., Cinderby S., Cambridge H., Evans P., Hill J.K. (2011). Habitat re-creation strategies for promoting adaptation of species to climate change. Conserv. Lett..

[119] Williams S.H., Scriven S.A., Burslem D.F.R.P., Hill J.K., Reynolds G., Agama A.L., Kugan F., Maycock C.R., Khoo E., Hastie A.Y.L. (2019). Incorporating connectivity into conservation planning for optimal representation of multiple species and ecosystem services. Conserv. Biol..

