# Supplementary material for: Predicting Hotspots and Prioritizing Protected Areas for Endangered Primate Species in Indonesia under Changing Climate
Source: Biology (Basel). 2021 Feb 15;10(2):154. doi: 10.3390/biology10020154 (PMC7919460; doi:10.3390/biology10020154)
Supplement: Supplementary file 1 [file biology-10-00154-s001.pdf]

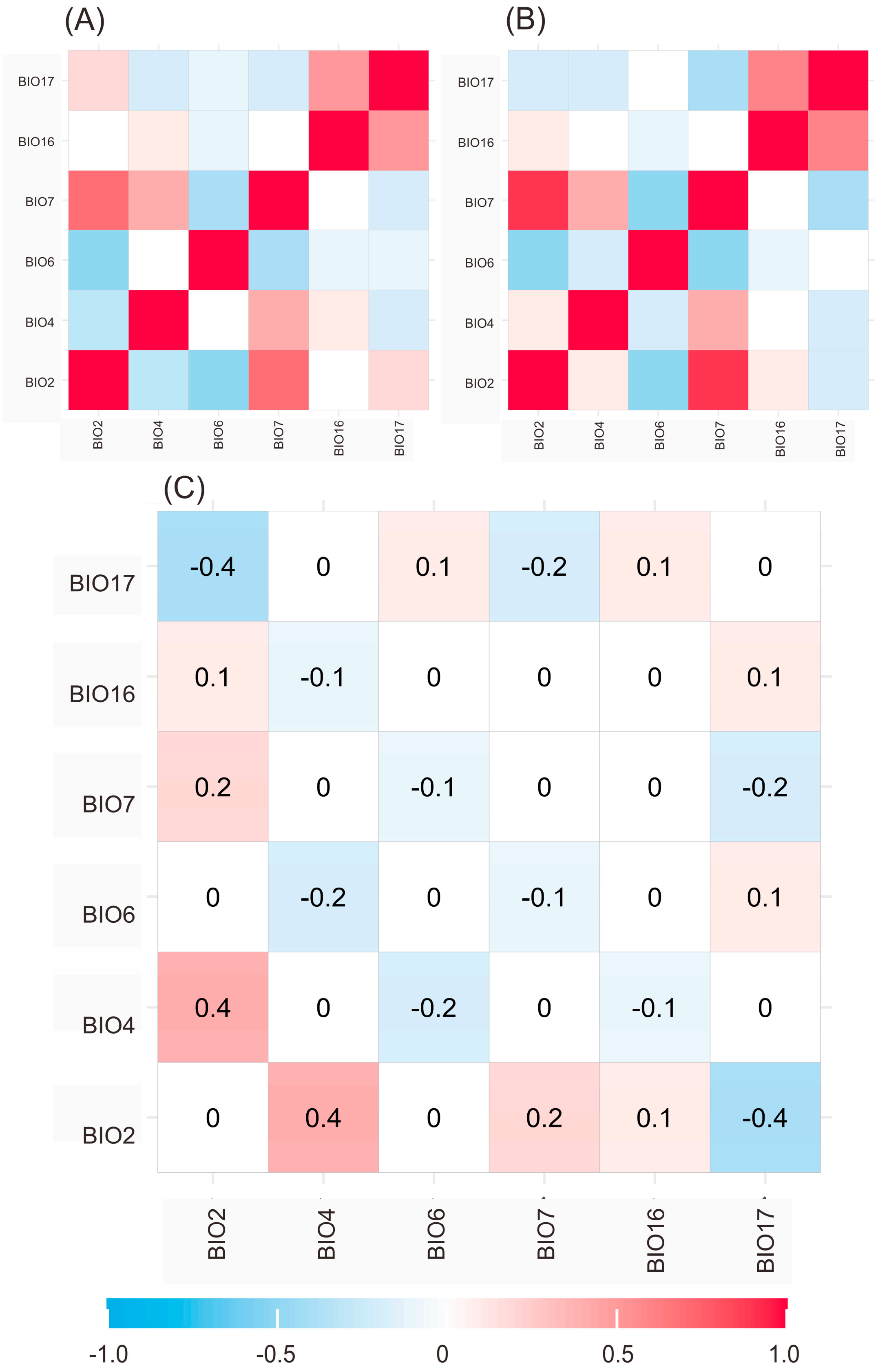

Figure S1. Degree of collinearity for the model training under current (A) and future (B) climate conditions using variables selection ($|r| \leq 0.7$). Collinearity shift between training and projected environments (C). In this study, we did not find any issues in the degree of collinearity for each climatic conditions and collinearity shift after variables selection.

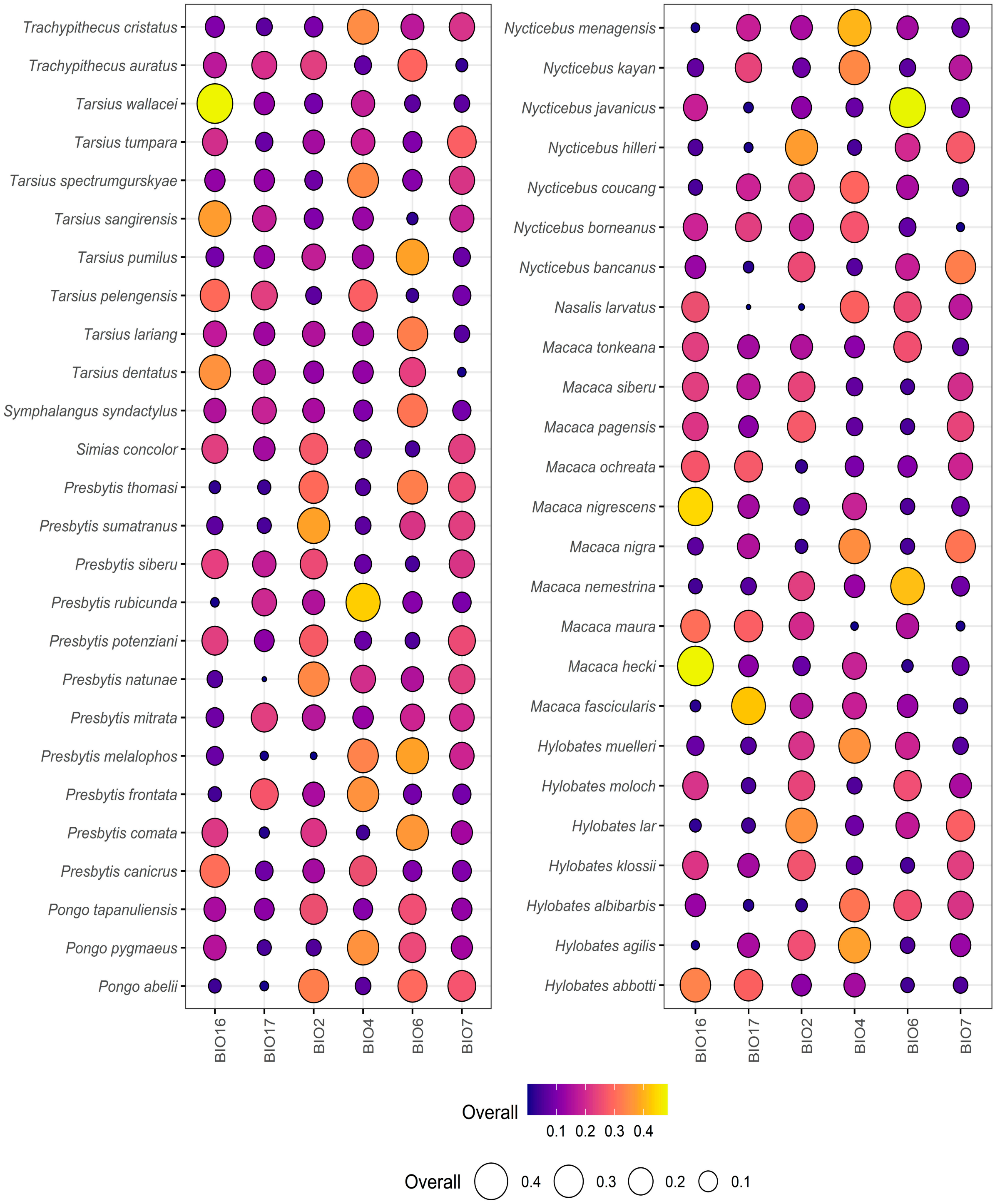

Figure S2. Variable importance (VI) scores for the potential distribution model of each Primates species. Big and red to yellow circles indicate a higher dependence of the species occurrence on the corresponding bioclimatic covariate. In the other hand, small and purple to blue circles indicate low dependence. The environmental covariates used in the models were, BIO16: precipitation of the wettest quarter, BIO17: precipitation of the driest quarter, BIO2: annual mean diurnal range of temperature, BIO4: temperature seasonality, BIO6: minimum temperature of the coldest month, and BIO7: annual temperature range.

Table S1. Predictive performance of the models on the k-fold (k = 5) for each species using five different metrics of accuracy, show in mean of value ± SE – i.e., Area Under the ROC Curve (AUC), Kappa coefficient, True Skill Statistic (TSS), Jaccard Index, and Sørensen Index.

Species	AUC	Kappa	TSS	Jaccard	Sørensen
Hylobates abbotti	0.98 ± 0.000	0.89 ± 0.003	0.89 ± 0.003	0.90 ± 0.003	0.94 ± 0.001
Hylobates agilis	0.91 ± 0.001	0.69 ± 0.003	0.69 ± 0.003	0.76 ± 0.002	0.86 ± 0.001
Hylobates albibarbis	0.61 ± 0.107	0.56 ± 0.101	0.56 ± 0.101	0.68 ± 0.058	0.79 ± 0.039
Hylobates klossii	0.76 ± 0.038	0.62 ± 0.038	0.62 ± 0.038	0.72 ± 0.022	0.83 ± 0.015
Hylobates lar	0.94 ± 0.002	0.79 ± 0.007	0.79 ± 0.007	0.83 ± 0.006	0.90 ± 0.003
Hylobates moloch	0.90 ± 0.002	0.76 ± 0.007	0.76 ± 0.007	0.79 ± 0.005	0.87 ± 0.003
Hylobates muelleri	0.95 ± 0.000	0.88 ± 0.001	0.88 ± 0.001	0.89 ± 0.001	0.94 ± 0.001
Macaca fascicularis	0.85 ± 0.001	0.64 ± 0.003	0.64 ± 0.003	0.68 ± 0.002	0.81 ± 0.001
Macaca hecki	0.98 ± 0.000	0.96 ± 0.002	0.96 ± 0.002	0.96 ± 0.002	0.98 ± 0.001
Macaca maura	0.84 ± 0.012	0.74 ± 0.015	0.74 ± 0.015	0.79 ± 0.012	0.88 ± 0.008
Macaca nemestrina	0.75 ± 0.053	0.66 ± 0.059	0.66 ± 0.059	0.75 ± 0.044	0.84 ± 0.030
Macaca nigra	0.93 ± 0.006	0.82 ± 0.011	0.82 ± 0.011	0.84 ± 0.009	0.91 ± 0.006
Macaca nigrescens	0.97 ± 0.002	0.88 ± 0.005	0.88 ± 0.005	0.89 ± 0.004	0.94 ± 0.002
Macaca ochreata	0.97 ± 0.001	0.88 ± 0.004	0.88 ± 0.004	0.89 ± 0.004	0.94 ± 0.002
Macaca pagensis	0.97 ± 0.001	0.87 ± 0.003	0.87 ± 0.003	0.88 ± 0.002	0.93 ± 0.001
Macaca siberu	0.99 ± 0.001	0.97 ± 0.000	0.97 ± 0.000	0.97 ± 0.000	0.98 ± 0.000
Macaca tonkeana	0.95 ± 0.001	0.78 ± 0.004	0.78 ± 0.004	0.80 ± 0.002	0.89 ± 0.002
Nasalis larvatus	0.96 ± 0.009	0.93 ± 0.018	0.93 ± 0.018	0.93 ± 0.018	0.96 ± 0.010
Nycticebus bancanus	0.99 ± 0.000	0.95 ± 0.006	0.95 ± 0.006	0.95 ± 0.005	0.97 ± 0.003
Nycticebus borneanus	0.92 ± 0.000	0.70 ± 0.001	0.70 ± 0.001	0.75 ± 0.001	0.86 ± 0.001
Nycticebus coucang	0.88 ± 0.001	0.64 ± 0.003	0.64 ± 0.003	0.72 ± 0.003	0.83 ± 0.002
Nycticebus hilleri	0.97 ± 0.000	0.90 ± 0.003	0.90 ± 0.003	0.90 ± 0.002	0.95 ± 0.001
Nycticebus javanicus	0.95 ± 0.024	0.93 ± 0.036	0.93 ± 0.036	0.93 ± 0.036	0.96 ± 0.022
Nycticebus kayan	0.94 ± 0.001	0.81 ± 0.000	0.81 ± 0.000	0.84 ± 0.000	0.91 ± 0.000
Nycticebus menagensis	0.81 ± 0.001	0.56 ± 0.002	0.56 ± 0.002	0.68 ± 0.002	0.81 ± 0.001
Pongo abelii	0.99 ± 0.002	0.94 ± 0.011	0.94 ± 0.011	0.95 ± 0.009	0.97 ± 0.005
Pongo pygmaeus	0.90 ± 0.015	0.73 ± 0.034	0.73 ± 0.034	0.80 ± 0.022	0.88 ± 0.014
Pongo tapanuliensis	0.99 ± 0.000	0.98 ± 0.002	0.98 ± 0.002	0.98 ± 0.002	0.99 ± 0.001
Presbytis canicrus	0.98 ± 0.000	0.91 ± 0.001	0.91 ± 0.001	0.92 ± 0.001	0.95 ± 0.001
Presbytis comata	0.93 ± 0.024	0.88 ± 0.038	0.88 ± 0.038	0.88 ± 0.038	0.93 ± 0.023
Presbytis frontata	0.88 ± 0.000	0.65 ± 0.002	0.65 ± 0.002	0.73 ± 0.001	0.84 ± 0.001
Presbytis melalophos	0.85 ± 0.032	0.76 ± 0.033	0.76 ± 0.033	0.77 ± 0.033	0.86 ± 0.020
Presbytis mitrata	0.95 ± 0.001	0.80 ± 0.004	0.80 ± 0.004	0.82 ± 0.003	0.90 ± 0.002
Presbytis natunae	0.94 ± 0.001	0.82 ± 0.002	0.82 ± 0.002	0.86 ± 0.001	0.91 ± 0.001
Presbytis potenziani	0.99 ± 0.000	0.99 ± 0.003	0.99 ± 0.003	0.99 ± 0.003	0.99 ± 0.002
Presbytis rubicunda	0.88 ± 0.002	0.66 ± 0.003	0.66 ± 0.003	0.72 ± 0.002	0.84 ± 0.001
Presbytis siberu	0.94 ± 0.002	0.82 ± 0.004	0.82 ± 0.004	0.86 ± 0.003	0.91 ± 0.002
Presbytis sumatranus	0.98 ± 0.000	0.91 ± 0.002	0.91 ± 0.002	0.91 ± 0.002	0.95 ± 0.001
Presbytis thomasi	0.94 ± 0.001	0.84 ± 0.002	0.84 ± 0.002	0.87 ± 0.002	0.92 ± 0.001
Simias concolor	0.95 ± 0.001	0.84 ± 0.004	0.84 ± 0.004	0.87 ± 0.003	0.92 ± 0.002
Symphalangus syndactylus	0.91 ± 0.034	0.86 ± 0.051	0.86 ± 0.051	0.86 ± 0.051	0.92 ± 0.030
Tarsius dentatus	0.98 ± 0.001	0.91 ± 0.005	0.91 ± 0.005	0.91 ± 0.005	0.95 ± 0.002
Tarsius lariang	0.94 ± 0.013	0.89 ± 0.021	0.89 ± 0.021	0.89 ± 0.021	0.93 ± 0.013
Tarsius pelengensis	0.96 ± 0.005	0.90 ± 0.010	0.90 ± 0.010	0.90 ± 0.010	0.94 ± 0.006
Tarsius pumilus	0.98 ± 0.001	0.90 ± 0.003	0.90 ± 0.003	0.91 ± 0.003	0.95 ± 0.002
Tarsius sangirensis	0.96 ± 0.005	0.90 ± 0.009	0.90 ± 0.009	0.90 ± 0.009	0.94 ± 0.005

Species	AUC	Kappa	TSS	Jaccard	Sørensen
Tarsius spectrumgurskyae	0.98 ± 0.002	0.94 ± 0.010	0.94 ± 0.010	0.95 ± 0.009	0.97 ± 0.005
Tarsius tumpara	0.99 ± 0.001	0.97 ± 0.010	0.97 ± 0.010	0.97 ± 0.009	0.98 ± 0.005
Tarsius wallacei	0.99 ± 0.000	0.97 ± 0.000	0.97 ± 0.000	0.97 ± 0.000	0.98 ± 0.000
Trachypithecus auratus	0.97 ± 0.003	0.89 ± 0.012	0.89 ± 0.012	0.89 ± 0.010	0.94 ± 0.006
Trachypithecus cristatus	0.86 ± 0.028	0.70 ± 0.055	0.70 ± 0.055	0.78 ± 0.033	0.87 ± 0.021

Table S2. Species-specific information of primate occurrences across Indonesia as response variable in the species distribution models. DD: Data Deficient; VU: Vulnerable; EN: Endangered; and CR: Critically Endangered.

Family	Species	IUCN status	Number of occurrences	Basis of Records
Cercopithecidae	*Macaca fascicularis*	VU	417	Human observation
	Macaca hecki	VU	25	Human observation
	Macaca maura	EN	100	Human observation
	Macaca nemestrina	EN	16	Human observation
	Macaca nigra	CR	39	Human observation
	Macaca nigrescens	VU	14	Human observation
	Macaca ochreata	VU	25	Human observation
	Macaca pagensis	CR	19	Human observation
	Macaca siberu	EN	18	Human observation
	Macaca tonkeana	VU	40	Human observation
	Nasalis larvatus	EN	27	Human observation
	Presbytis canicrus	EN	60	Human observation
	Presbytis comata	EN	18	Human observation
	Presbytis frontata	VU	80	Human observation
	Presbytis melalophos	EN	17	Human observation
	Presbytis mitrata	VU	40	Human observation
	Presbytis natunae	VU	98	Human observation
	Presbytis potenziani	CR	18	Human observation
	Presbytis rubicunda	VU	68	Human observation
	Presbytis siberu	EN	19	Human observation
	Presbytis sumatranus	EN	68	Human observation
	Presbytis thomasi	VU	56	Human observation
	Simias concolor	CR	28	Human observation
	Trachypithecus auratus	VU	46	Human observation
	Trachypithecus cristatus	VU	19	Human observation
Hominidae	*Pongo abelii*	CR	57	Human observation
	Pongo pygmaeus	CR	49	Human observation
	Pongo tapanuliensis	CR	40	Human observation
Hylobatidae	*Hylobates abbotti*	EN	43	Human observation
	Hylobates agilis	EN	80	Human observation
	Hylobates albibarbis	EN	13	Human observation
	Hylobates klossii	EN	27	Human observation
	Hylobates lar	EN	10	Human observation
	Hylobates moloch	EN	26	Human observation

Family	Species	IUCN status	Number of occurrences	Basis of Records
	Hylobates muelleri	EN	65	Human observation
	Symphalangus syndactylus	EN	13	Human observation
Lorisidae	*Nycticebus bancanus*	CR	28	Human observation
	Nycticebus borneanus	VU	79	Human observation
	Nycticebus coucang	EN	88	Human observation
	Nycticebus hilleri	EN	55	Human observation
	Nycticebus javanicus	CR	17	Human observation
	Nycticebus kayan	VU	59	Human observation
	Nycticebus menagensis	VU	67	Human observation
Tarsidae	*Tarsius dentatus*	VU	40	Human observation
	Tarsius lariang	DD	25	IUCN species range
	Tarsius pelengensis	EN	47	Human observation
	Tarsius pumilus	DD	30	IUCN species range
	Tarsius sangirensis	EN	47	Human observation
	Tarsius spectrumgurskyae	VU	23	Human observation
	Tarsius tumpara	CR	42	Human observation
	Tarsius wallacei	DD	24	IUCN species range

Table S3. Global climate models used in ecological niche modeling projections for 2050 based on RCP4.5 and RCP8.5 scenarios.

Abbreviated Model Name	Institution	Source
ACCESS1-0	Commonwealth Scientific and Industrial Research Organisation (CSIRO) and Bureau of Meteorology (BoM)	[1]
BCC-CSM1-1	Beijing Climate Center Climate System Model	[2]
CCSM4	University Corporation for Atmospheric Research (UCAR)	[3]
CESM1-CAM5-1-FV2	National Center for Atmospheric Research (NCAR)	[4]
CNRM-CM5	Centre National de Recherches Météorologiques	[5]
GFDL-CM3	NOAA Geophysical Fluid Dynamics Laboratory	[6]
GFDL-ESM2G	NOAA Geophysical Fluid Dynamics Laboratory	[7]
GISS-E2-R	NASA Goddard Institute for Space Studies USA	[8]
HadGEM2-AO	Met Office Hadley Centre UK	[9]
HadGEM2-CC	Met Office Hadley Centre UK	[10]
HadGEM2-ES	Met Office Hadley Centre UK	[11]
INMCM4	Russian Institute for Numerical Mathematics Climate Model	[12]
IPSL-CM5A-LR	Institut Pierre Simon Laplace	[13]
MIROC-ESM-CHEM	University of Tokyo, National Institute for Environmental Studies, and Japan Agency for Marine-Earth Science and Technology	[14]
MIROC-ESM	University of Tokyo, National Institute for Environmental Studies, and Japan Agency for Marine-Earth Science and Technology	[14]
MIROC5	University of Tokyo, National Institute for Environmental Studies, and Japan Agency for Marine-Earth Science and	[15]

Abbreviated Model Name	Institution	Source
	Technology	
MPI-ESM-LR	Max-Planck-Institut für Meteorologie	[16]
MRI-CGCM3	Meteorological Research Institute	[17]
NorESM1-M	Norwegian Climate Center's Earth System Model	[18]

References

1. Bi, D.; Dix, M.; Marsland, S.J.; O'Farrell, S.; Rashid, H.A.; Uotila, P.; Hirst, A.C.; Kowalczyk, E.; Golebiewski, M.; Sullivan, A.; et al. The ACCESS coupled model: Description, control climate and evaluation. *Aust. Meteorol. Oceanogr. J.* **2013**, *63*, 41–64, doi:10.22499/2.6301.004.
2. Wu, T.; Song, L.; Li, W.; Wang, Z.; Zhang, H.; Xin, X.; Zhang, Y.; Zhang, L.; Li, J.; Wu, F.; et al. An overview of BCC climate system model development and application for climate change studies. *J. Meteorol. Res.* **2014**, *28*, 34–56, doi:10.1007/s13351-014-3041-7.
3. Gent, P.R.; Danabasoglu, G.; Donner, L.J.; Holland, M.M.; Hunke, E.C.; Jayne, S.R.; Lawrence, D.M.; Neale, R.B.; Rasch, P.J.; Vertenstein, M.; et al. The community climate system model version 4. *J. Clim.* **2011**, *24*, 4973–4991, doi:10.1175/2011JCLI4083.1.
4. Meehl, G.A.; Washington, W.M.; Arblaster, J.M.; Hu, A.; Teng, H.; Kay, J.E.; Gettelman, A.; Lawrence, D.M.; Sanderson, B.M.; Strand, W.G. Climate change projections in CESM1(CAM5) compared to CCSM4. *J. Clim.* **2013**, *26*, 6287–6308, doi:10.1175/JCLI-D-12-00572.1.
5. Voldoire, A.; Sanchez-Gomez, E.; Salas y Mélia, D.; Decharme, B.; Cassou, C.; Sénési, S.; Valcke, S.; Beau, I.; Alias, A.; Chevallier, M.; et al. The CNRM-CM5.1 global climate model: Description and basic evaluation. *Clim. Dyn.* **2013**, *40*, 2091–2121, doi:10.1007/s00382-011-1259-y.
6. Griffies, S.M.; Winton, M.; Donner, L.J.; Horowitz, L.W.; Downes, S.M.; Farneti, R.; Gnanadesikan, A.; Hurlin, W.J.; Lee, H.C.; Liang, Z.; et al. The GFDL CM3 coupled climate model: Characteristics of the ocean and sea ice simulations. *J. Clim.* **2011**, *24*, 3520–3544, doi:10.1175/2011JCLI3964.1.
7. Dunne, J.P.; John, J.G.; Adcroft, A.J.; Griffies, S.M.; Hallberg, R.W.; Shevliakova, E.; Stouffer, R.J.; Cooke, W.; Dunne, K.A.; Harrison, M.J.; et al. GFDL's ESM2 global coupled climate-carbon earth system models. Part I: Physical formulation and baseline simulation characteristics. *J. Clim.* **2012**, *25*, 6646–6665, doi:10.1175/JCLI-D-11-00560.1.
8. Schmidt, G.A.; Kelley, M.; Nazarenko, L.; Ruedy, R.; Russell, G.L.; Aleinov, I.; Bauer, M.; Bauer, S.E.; Bhat, M.K.; Bleck, R.; et al. Configuration and assessment of the GISS ModelE2 contributions to the CMIP5 archive. *J. Adv. Model. Earth Syst.* **2014**, *6*, 141–184, doi:10.1002/2013MS000265.Received.
9. Baek, H.J.; Lee, J.; Lee, H.S.; Hyun, Y.K.; Cho, C.; Kwon, W.T.; Marzin, C.; Gan, S.Y.; Kim, M.J.; Choi, D.H.; et al. Climate change in the 21st century simulated by HadGEM2-AO under representative concentration pathways. *Asia-Pacific J. Atmos. Sci.* **2013**, *49*, 603–618, doi:10.1007/s13143-013-0053-7.
10. Martin, G.M.; Bellouin, N.; Collins, W.J.; Culverwell, I.D.; Halloran, P.R.; Hardiman, S.C.; Hinton, T.J.; Jones, C.D.; McDonald, R.E.; McLaren, A.J.; et al. The HadGEM2 family of Met Office Unified Model climate configurations. *Geosci. Model Dev.* **2011**, *4*, 723–757, doi:10.5194/gmd-4-723-2011.
11. Jones, C.D.; Hughes, J.K.; Bellouin, N.; Hardiman, S.C.; Jones, G.S.; Knight, J.; Liddicoat, S.; O'Connor, F.M.; Andres, R.J.; Bell, C.; et al. The HadGEM2-ES implementation of CMIP5 centennial simulations. *Geosci. Model Dev.* **2011**, *4*, 543–570, doi:10.5194/gmd-4-543-2011.
12. Volodin, E.M.; Dianskii, N.A.; Gusev, A. V. Simulating present-day climate with the INMCM4.0 coupled model of the atmospheric and oceanic general circulations. *Izv. - Atmos. Ocean Phys.* **2010**, *46*, 414–431, doi:10.1134/S000143381004002X.
13. Dufresne, J.L.; Foujols, M.A.; Denvil, S.; Caubel, A.; Marti, O.; Aumont, O.; Balkanski, Y.; Bekki, S.; Bellenger, H.; Benshila, R.; et al. *Climate change projections using the IPSL-CM5 Earth System Model: From CMIP3 to CMIP5*; 2013; Vol. 40; ISBN 0038201216.
14. Watanabe, S.; Hajima, T.; Sudo, K.; Nagashima, T.; Takemura, T.; Okajima, H.; Nozawa, T.; Kawase, H.; Abe, M.; Yokohata, T.; et al. MIROC-ESM 2010: Model description and basic results of CMIP5-20c3m experiments. *Geosci. Model Dev.* **2011**, *4*, 845–872, doi:10.5194/gmd-4-845-2011.
15. Tatebe, H.; Ogura, T.; Nitta, T.; Komuro, Y.; Ogochi, K.; Takemura, T.; Sudo, K.; Sekiguchi, M.; Abe, M.; Saito, F.; et al. Description and basic evaluation of simulated mean state, internal variability, and climate sensitivity in MIROC6. *Geosci. Model Dev.* **2019**, *12*, 2727–2765, doi:10.5194/gmd-12-2727-2019.
16. Block, K.; Mauritsen, T. Forcing and feedback in the MPI-ESM-LR coupled model under abruptly quadrupled CO2. *J. Adv. Model. Earth Syst.* **2013**, *5*, 676–691, doi:10.1002/jame.20041.

17. Yukimoto, S.; Adachi, Y.; Hosaka, M.; Sakami, T.; Yoshimura, H.; Hirabara, M.; Tanaka, T.Y.; Shindo, E.; Tsujino, H.; Deushi, M.; et al. A new global climate model of the Meteorological Research Institute: MRI-CGCM3: Model description and basic performance. *J. Meteorol. Soc. Japan* **2012**, *90*, 23–64, doi:10.2151/jmsj.2012-A02.
18. Iversen, T.; Bentsen, M.; Bethke, I.; Debernard, J.B.; Kirkevåg, A.; Seland, Ø.; Drange, H.; Kristjansson, J.E.; Medhaug, I.; Sand, M.; et al. The Norwegian Earth System Model, NorESM1-M – Part 2: Climate response and scenario projections. *Geosci. Model Dev.* **2013**, *6*, 389–415, doi:10.5194/gmd-6-389-2013.
